# Colorizing Grayscale CT images of human lungs using deep learning methods

**DOI:** 10.1007/s11042-022-13062-0

**Published:** 2022-04-22

**Authors:** Yuewei Wang, Wei Qi Yan

**Affiliations:** Auckland University of Technology, Auckland, 1010 New Zealand

**Keywords:** Colorization, Deep learning, CNN, Lung CT images

## Abstract

Image colorization refers to computer-aided rendering technology which transfers colors from a reference color image to grayscale images or video frames. Deep learning elevated notably in the field of image colorization in the past years. In this paper, we formulate image colorization methods relying on exemplar colorization and automatic colorization, respectively. For hybrid colorization, we select appropriate reference images to colorize the grayscale CT images. The colours of meat resemble those of human lungs, so the images of fresh pork, lamb, beef, and even rotten meat are collected as our dataset for model training. Three sets of training data consisting of meat images are analysed to extract the pixelar features for colorizing lung CT images by using an automatic approach. Pertaining to the results, we consider numerous methods (i.e., loss functions, visual analysis, PSNR, and SSIM) to evaluate the proposed deep learning models. Moreover, compared with other methods of colorizing lung CT images, the results of rendering the images by using deep learning methods are significantly genuine and promising. The metrics for measuring image similarity such as SSIM and PSNR have satisfactory performance, up to 0.55 and 28.0, respectively. Additionally, the methods may provide novel ideas for rendering grayscale X-ray images in airports, ferries, and railway stations.

## Introduction

Since the end of 2019, the COVID-19 epidemic has thoroughly broken out across the world, which generates adverse influences beyond the imagination of public. For example, our ordinary activities are restricted due to the COVID-19 remarkably. Up to date, more than 188 million confirmed cases in the whole world. All clinical trials reflect that human lungs are fundamentally affected and damaged by the coronavirus. Computed Tomography (CT) is applied to diagnostic examinations, which was previously called Computed Axial Tomography [5]. Although CT applies X-ray, lung CT images are formed through computer calculations. At present, professional doctors adjust the positions of a window to enhance the image contrast of CT images, which assists medics to visibly distinguish complex tissues in the grayscale image to increase the success rate of diagnosis. Colour CT images positively influence doctors’ efficiency and accuracy while observing the CT images to diagnose the diseases. As a fact, it is not only for doctors to check the status of the inertial organ but also for patients to comprehend the exact condition of their bodies without any professional knowledge by simply inspecting the lung CT images. Thus, lung CT images in grayscale have not full medical information to present the condition of organs. Therefore, we expect to colorize the CT images that could show the colours as realistic and vivid as our human tissues.

A plenty of studies have been summarized that contagious lungs are split into a total of four stages according to the degree of lung damage through the given COVID-19 lung CT images: Early stage, progressive stage, peak stage, and absorption stage [22]. These stages reveal that COVID-19 has various scopes and extent of influence as the condition of an infected patient becomes worsened. Normally, the texture of CT images of infected lungs is more complicated than that of healthy lung CT images [10]. A considerable number of lung CT images have been uploaded to the Internet. The lung CT images in color were generated by using different approaches. In this paper, deep neural networks are considered as the most straightforward method to render the grayscale images of our human tissues in the computational domain. We believe deep neural networks have the ability to fulfil the colourizing work. Thus, we think that colorization methods in the domain of deep learning would be a brave endeavour for colorizing the lung CT images in grayscale.

The foremost research question of this article is to colorize grayscale lung CT images with two distinct approaches in the field of colorization from deep learning. In order to accomplish much reliable performance in these ultimately generated lung CT images, we propose two essential approaches to generate colour lung CT images. The initial one is to assemble a diversity of training datasets to automatically colorize the grayscale lung CT images by using ResNet model [31]. Simultaneously, another method was proposed to utilize various reference images to transfer the style and content of colors to the target lung CT images through VGG-19 network [2]. VGG-19 and ResNet are both implemented by using Convolution Neural Networks (CNNs or ConvNets). The ultimate consequences are essentially compared via human visual system and measured by using the metrics from full-reference methods. Pertaining to the target images, we merely select the lung CT images from two stages of COVID-19 infection in this paper. Moreover, the lung CT images from healthy people are additionally collected in our project to obtain the appropriate colorization methods.

The remaining parts of this paper encapsulate literature review in Section 2, our methods in Sections 3 and 4, our results Section 5, our conclusion and future work will be depicted in Section 6.

## Literature review

In this paper, we would like to fully review the literature in image colorization for lung CT images in grayscale. With a thoughtful review of the previous work, we notice that there are four approaches to explore the problem of image colorization, including scribble-based colorization, exemplar-based colorization, machine learning and deep learning-based colorization, and hybrid methods of colorization. Owing to the colorizing lung CT images being involved in multiple domains, we implement the colorizing methods, namely, CNN-based colorization method and hybrid colorization methods in our experiments.

### Scribble-based image colorization

Scribble-based image colorization was a time-consuming and expensive task which normally is implemented through manual operations by using Adobe Photoshop (PS) and other software. Therefore, it is also named as human-labelled color scribbles. One picture usually demands a multitude of weeks to be colorized, for instance, human face processing solely requires up to 20 layers of pink, green, and blue shades to be recolourised correctly [23].

YUV colour space is proposed which provided the basic knowledge of colorization, where Y indicates the monochromatic luminance channel (simply named as intensity), U and V are defined as the chrominance channels and encoding the colour, respectively [18]. The contiguous pixels with similar luminance have a similar colour for generating rare scribble colours and the final result has obviously high performance [18]. The specific operations are employed to scribble the colours in an image and colorize the remaining blank space in an optimization method [18, 26, 33]. The structural characteristics of textures have been considered profoundly to implement the highest frequency in the approach of scribble-based colorization [6, 11, 20, 21, 24, 25, 30].

The scribble-based colorization is the first step to investigate and discover the methodology in the colorization of grayscale images and videos. However, the main drawback of these approaches is that the initialization of this colorization process is performed completely manually. Moreover, for providing good scribbles, labour intensive work and expert skills are extremely needed to colorize an image with fine-scale compositions. Meanwhile, it also spends extra cost of computations to obtain high-quality outcomes.

### Exemplar-based image colorization

Exemplar-based image colorization has a rigorous prerequisite to select reference color images, which were captured from the scenes with the same background as the grayscale target images. This method is also called as patch-based colorization. The early method is based on a form of color revision that renders the target images from a reference image by using a simple statistical analysis [25]. The similar work has been emerged in 2002, where specific procedure is to transfer merely chromatic information through matching colors and maintaining the original luminance of the target images [30]. The performance is heightened by using the coordinating regions of the two images. However, since this approach has neglected the information of individual pixels, it produced disappointing outcomes. Gradually, more accurate color transfer is accomplished, including segmented regions [6, 9, 15, 27] superpixel [4, 9, 12, 19] and image retrieval methods [9, 19].

A segmented image will be applied to maximize the probability of each pixel of the entire colorized image for colorizing grayscale images at the global level. Compared to the methods of automatic color transfer, this approach exhibits the high performance of spatial consistency. More effective methods have been discovered in machine learning that will obtain color information from a set of drawings. The expectation-maximization (EM) and Gaussian mixture model (GMM) were employed for this rendering. The former is to induce natural connectivity among pixels by enhancing both spatial and color smoothness, the latter is to distinguish the color statistics in each domain [27]. The perceptually-alike semantic structures are taken into consideration in the algorithms of color transfer to utilize neural representations for matching spatial variance and global coherence [13].

This method of image colorization provides a much spontaneous approach to minimize the amount of human labor by supplying an extremely similar reference to the input grayscale images. However, the drawback of this method is that the performance of the target image extremely relies on the selected reference images. Moreover, a number of exemplar-based colorization methods have the assumption that the pixels with similar intensities or within similar regions should have comparable colors.

### Machine learning and deep learning-based image colorization

Machine learning and deep learning methods have been extensively implemented to render the grayscale images and videos in the past few years. Specifically, a fully-automatic image colorization method relies on locating the numerous patch and pixels of similar images from a massive reference image, transferring colours from the matched block to the pixel of the final target image. A unique outcome is produced for each input [6].

Deep neural networks in image colorization yet are sufficiently explored before because deep learning is the most advanced method in recent years. The fundamental manner of deep learning applies CNNs to extract features automatically from the large-scale data and predict the possible colours of target image. Semantic information and colour histograms of each image spot that these two intuitive observations are reflected in the design. With the presupposition of an excellent patch-matching method, extremely large-scale reference data has been employed for model training, including joint bilateral filtering as a postprocessing step to eliminate artifacts offered by using CNN networks [8]. However, the noises of patch matching are liable to increase with the size of the applied data in inconstant training. As the early investigator, Zhang et al. employed CNN to colorize the grayscale images. They leveraged the deep neural network to attest the empirical probability distribution by predicting 313 sets of the gamut, converted into ‘a’ and ‘b’ channels of the ”Lab” colour model [35].

In the domain of image colorization by using deep learning, the models such as Inception, ResNet, and VGG nets are typically utilized in the experiments. Zhang et al. leveraged the Inception ResNetv2 network and retrieved the reference and target images rather than training a feature extraction model from the inception layer [34]. Throughout training the deep convolutional network model, colour histogram (hue and chroma distributions) of each pixel was employed for developing a fully automatic colorization method. An intriguing method was proposed [17], in which an entirely convolutional version of VGG-16 net without any classification layers was applied to create a colour probability distribution. There are eight blocks which are accumulated in the approach of linear convolutional stacks. Either two or three convolutional layers followed by a ReLU activation function and Batch Normalization (BatchNorm) layer incorporate one block. Similarly, our method also utilized linear activation functions, but 10 blocks are combined by using a series of Conv-Leaky-ReLU functions and a BatchNorm layer to configure the VGG-19 model. Striding is applied to decrease the size of the image in lieu of pooling. A nonlinear activation function (Leaky-ReLU activation) has been applied to image colorization [32].

### Hybrid methods for image colorization

The scribble-based image colorization methods are more robust than the machine learning-based methods. Zhang et al. (2017) proposed to develop a comparable model which utilizes an intuitive way that applies image retrieval to automatically accomplish the reference collection [36]. The hybrid of colorizing model via CNNs and Inception-ResNetv2 was employed by importing the inputs to the model [3]. The focus of these models is on neural networks by using 20 color images to render grayscale images and attain 30*%* satisfactory rate after the process of image colorization.

## CNN-based image colorization methods

### Preprocessing

#### Training data

Quality and quantity are the two ways to measure a dataset. We collect two sets of images with completely distinct content to explore the influence of dataset for rendering lung CT images. One of the datasets is not relevant to human organs, including 2,129 painting images from the Kaggle. Another dataset seems much suitable for colorizing lung CT images by using 2,100 color meat images, which is possible to obtain genuine outcomes after colorizing grayscale lung CT images. Additionally, we are curious whether the amount of the visual data affects the final outcomes. Therefore, we randomly selected 30 color meat images instead all of them.

#### Image preprocessing

Two necessary procedures are needed to deal with original images (training data) for rendering grayscale one to colour images. Initially, we convert an RGB image into an Lab image and separate the channels ‘L’, ‘a’, and ‘b’ from the image to train the model for predicting the values of ‘a’ and ‘b’. Secondly, we convert a RGB image into a LUV image, likewise, separate the values ‘L’, ‘U’, and ‘V’ from the training images and then configure the relevant model to obtain the values of ‘U’ and ‘V’. The trichromatic system remains the most straightforward way in our implementation. However, the computationally intensive RGB pixels are not the ideal selection for colour image processing. When a series of problems appear, such as the essential association between channels, perceptual nonlinearity with visual perception, device dependence, chrominance and luminance data mixing. Consequently, we seek CIE-Lab and CIE-LUV colour spaces that are decent for digital image processing and uniform perceptually.

### Architecture

**Fig. 1:**
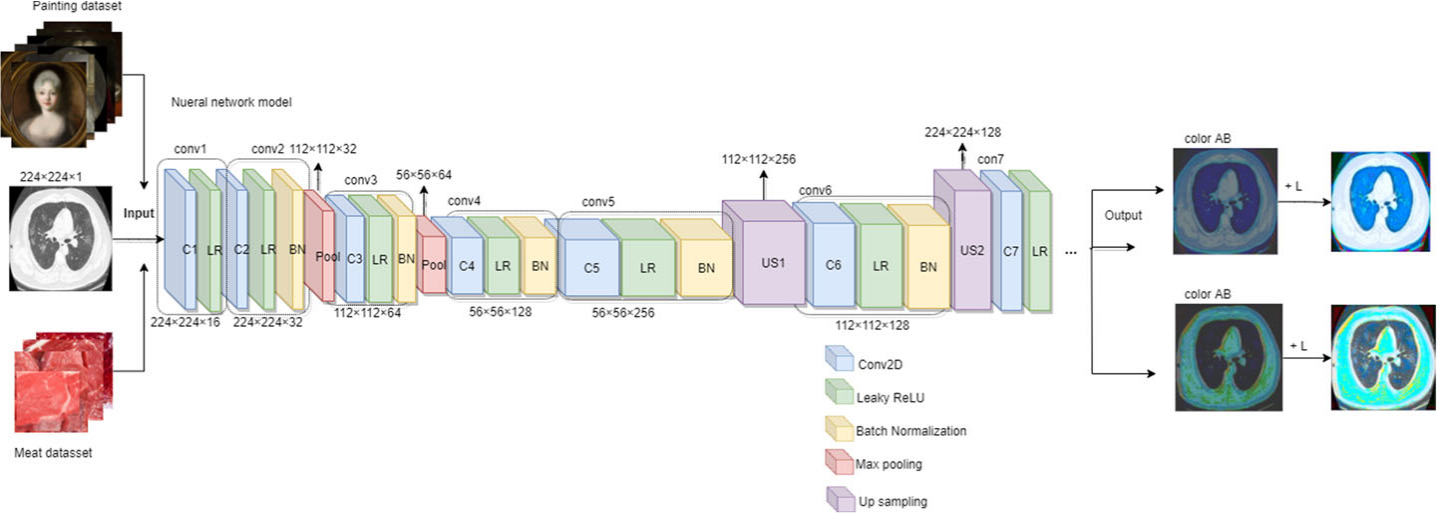
The architecture of CNN-based method for colorizing lung CT images in grayscale

The objective of this method is to implement the standard elementary autoencoder that carries out rendering lung CT images in grayscale. This full model is implemented with high-level or complicated methods to solve image colorization problem by employing TensorFlow [14]. The structure of our CNN-based image colorization model is presented in Fig. 1. The network linearly stacks convolutional layers by forming eight blocks, each block comprises either two or three convolutional layers followed by using a Leaky-ReLU layer and terminating in a Batch Normalization layer. Moreover, we apply upsampling to enlarge the number of feature maps in lieu of max pooling during the operations. The black and white layer signifies our input information; meanwhile, these two colours belong to the output layers. With the correlation between the grayscale images and the colourful training images, we decrease the size of the images to extract the features. For example, when we apply a 3 × 3 filter to per image in the dataset and combine the innovative pixels with the simplistic filters, the complicated patterns are able to be detected, such as a semicircle, a small point, a curve line, even a tiny straight line. Frequently, the model extracts the identical pixel from the images and then generates 128 unique purified images.

Generally, a neural network (filter or model) constitutes a link between inputs and output. The inputs refer to the lung CT grayscale images, the output indicates the numbers of filters and two-colour channels (‘a’ and ‘b’ in Lab colour model). The ultimate lung CT image is generated by combining these two filters. The original input lung CT images are adjusted to the size 224 × 224. The deep neural network inputs a grayscale image and predicts “ab” pairs of the gamut, reveals the empirical probability distribution, which is transformed to ‘a’ and ‘b’ channels of the Lab colour space. Eventually, it is upsampled to the original image size. Meanwhile, appending the lightness channel ‘L’ generates the lung CT images.

## Hybrid image colorization methods

### Architecture

In the method of hybrid colorization, the reference images are all collected by ourselves, we acquired images of fresh pork, lamb, steak from our supermarkets. Due to COVID-19 lung CT images in our project, considered the infected lungs, the meat we bought and took home without any processing to store for obtaining the rotten meat. Regarding the deep neural networks, the VGG net is taken the priority in this method. We eventually implemented the VGG-19 network to configure our colorization model. The VGG-19 works on convolutional networks to decompose reference images, which extracts associated styles and content for colorizing the grayscale CT images. Although the VGG-19 network has the functionality and principle as same as the VGG-16 network, the VGG-19 net demands more space to deal with images due to more convolutional layers and pooling layers. The activation function efficiently connects the input layer and the output layer [1].

**Fig. 2:**
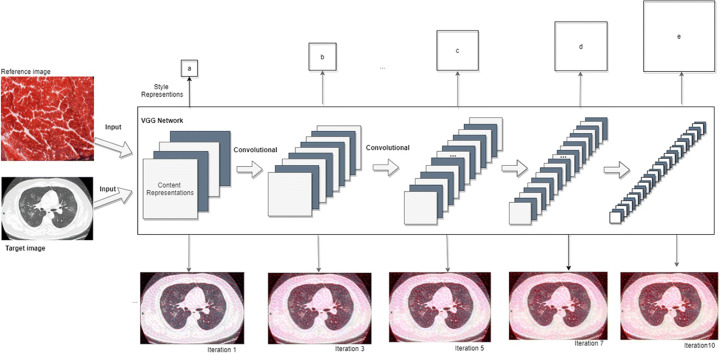
The iterative procedure of colour transfer from the reference image to the target image

With the hierarchy structure of deep neural networks, the input image is converted into a sequence that can be understood by computers. If the model is trained for object colorization, the target lung CT images are generated by transferring the object information gradually. The image is reconstructed by using feature maps so that the input image is visualized in each layer of the neural network. In Fig. 2, an input image is accepted to represent a set of filtered images at each stage of the neural network. The reconstruction is to restore the input image by simply comprehending the responses of each layer of the network so as to visualize the information of multiple stages in the model. In contrast, the reconstruction from lower layers will merely imitate the exact pixels of the original image. In order to obtain a representation of the input image, we apply a feature space initially designed to capture textural information, which is produced based on the filter response of each layer of the neural network. It consists of correlations between various responses within the spatial domain of the feature map. The image merely reflects its textural information rather than the global information. In Fig. 2, we use larger squares to present the styles.

In total, five basic blocks of convolutions are adopted in the model. In the first step, the input image is trained by applying VGG-19 to achieve the information of reference image for rendering the target image. The input image has the textural information and takes use of the filter responses of each layer network. We minimize the mean squared error of the the Gram matrices between the input image and the output image so as to obtain the final consequences. Specifically, the two sets of convolutional layers with 3 × 3 filters and 64 filters are adopted in the model. The height and width of images are 400 and 585, respectively, which terminates with the size 400× 585 ×64. Moreover, the convolutional kernel 64×2 indicates that the second convolutional layer with 64 filters. The filters are operated continuously with the kernel 3 × 3 with a stride of 1. A pooling layer that determines a volume will be diminished, the height and width of layers are spilled equally. The Gram matrix is adopted to measure the inconsistency of the target image and the reference image. Eventually, the style is transmitted and the ultimate images are generated by selecting the smallest variation in the background of the input image.

## Results and analysis

### Colorizing lung CT images using CNN-based method

In comparison to the lung CT images colorized by using three datasts, the CT images were rendered by using the painting works and 30 meat images, which has strong contrast to the dataset consisting of 2,100 meat images as shown in Table 1. The original RGB color images are decomposed into the three channels: Red, green and blue. Although the colors of the images seem merely to combine black and white colors together, three methods were applied to generate this kind of grayscale.

**Table 1:** The final results of the CNN-based method

	CT-lung (original)	Paintings data	Meat data	Meat data
		2,129 pics	2,100 pics	30 pics
COVID-19 lugn_01				
COVID-19 lugn_02				
Healthy lung				
Model iteration		epochs= 53,verbose= 1,st	epochs= 50,verbose= 1,st	epochs= 10,verbose= 1,st
parameters		eps_per_epoch= 38	eps_per_epoch= 42	eps_per_epoch= 3

**Fig. 3:**
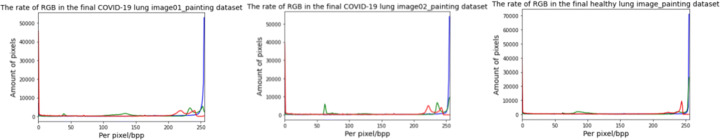
The final colorizing lung CT images with the painting dataset

**Fig. 4:**
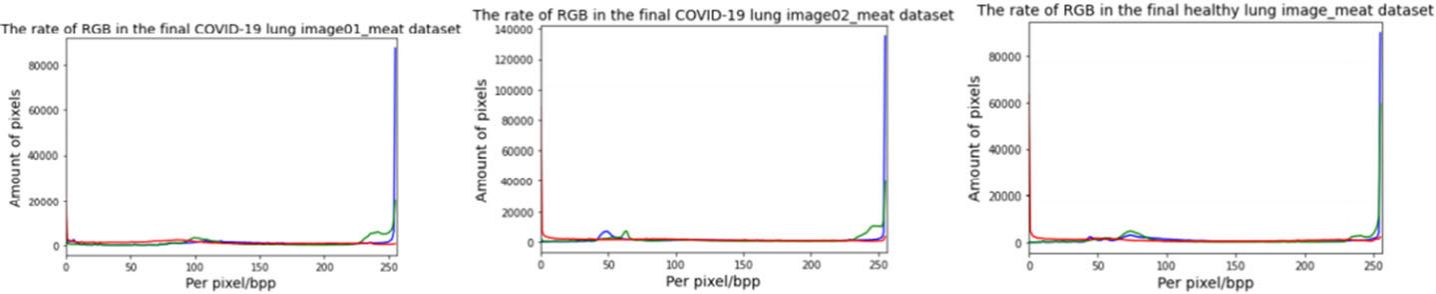
The final colorizing lung CT images with the meat dataset

Figures 3 and 4 show the proportion of RGB colors in the outcome images from the painting works and 30 meat images, respectively. From visual analysis, we believe that a training dataset performs more accurately than a small amount of data. In addition, compared to the two collections of images with paintings and meat images, the results by using these two sets exhibit the corresponding advantages. In the experiment by using the training set of painting works, we show the rendered lung CT images. By using other meat images, we discovered the differences between healthy lung CT images and infected lung CT images.

### Colorizing lung CT images using hybrid methods

#### Steak images as the reference

**Fig. 5:**
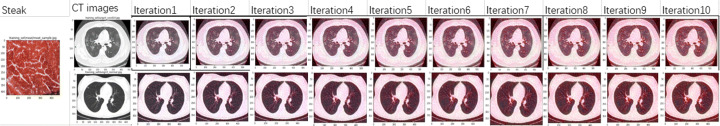
The results with hybrid method and steak images as the reference ones

The results in each iteration of lung CT images (COVID-19 lung and healthy lung) with steak images as the reference image is illustrated in Fig. 5. Apparently, the colours of generated images are gradually intensifying as the number of iterations increases. It shows that lung CT images are progressively being rendered owing to the deep neural networks. From the visual evaluation of experimental results, the colour deviation to the most standard red is the closest one to the colour of human organ. The original white part in the CT grayscale images has been replaced by using red color. Moreover, red is a frequently superimposed colour in the black part of grayscale lung CT images. On the whole, the lung CT images after colorization are extraordinarily colorful.

#### Lamb images as the reference

**Fig. 6:**
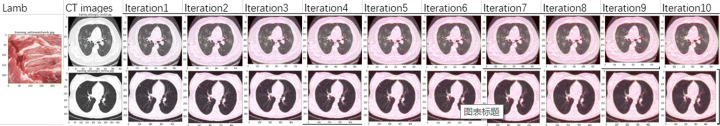
The hybrid method by using lamb images as the reference ones

According to the reference image colour, the lamb colours are more moderate than the colour of steak images in Fig. 6. Therefore, the coloured lung CT images generated by using lamb as a reference image are more balance than the steak image. Visually, the fat part of lamb is extended (white part), the generated target image is more accurate than the reference images.

#### Pork images as the reference

**Fig. 7:**
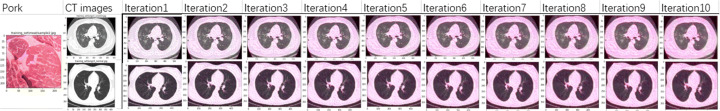
The hybrid method by using images of pork as reference one

The reference colours of pork images are essentially pinkish, the ultimately generated images are also approximately pink in Fig. 7. From the results of lung CT images colorizing, we realize that pink is an evenly bloomed colour in the reference image. Because there is no gradual colours, the details of the lung CT images are possible not to perform as robust as other reference images.

#### Rotten meat images as the reference

**Fig. 8:**
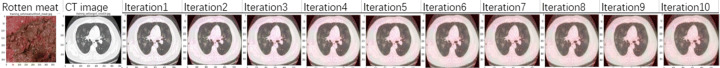
The hybrid method by using images of rotting meat as reference ones

For decaying meat images as a reference to colorize the infected lung CT images, we observe that the colours of the entire lung images have attained the superior result in Fig. 8. The colours of rotten flesh are composed of brown and red. Consequently, the infected lung CT images are evenly colourized by these two colours. It confirms that our proposed model adequately transfers the colour components of the reference images to the target images effectively.

It is challenging to differentiate visual observations which one of these reference images presents a dependable outcome. We discerned that the fat part of lamb images forms a particular advantage compared to others as the reference. We noticed the infected lung images that the rotten meat images as the reference also produced a remarkable outcome. The model is fully employed to render the entire lung CT images. Meanwhile, it also demonstrates that the supposition of this model is remarkably effective.

### Evaluation methods

The quality of CT images depends on four fundamental factors: Image contrast, spatial resolution, image noises and artefacts [28]. PSNR (i.e., peak signal-to-noise ratio) is customarily employed to estimate and compare the quality between the reconstructed image and the original image. The evaluation of structural SIMilarity (SSIM) metric consolidates the original contrast of images, luminance, and structure as a unique local property score. Moreover, PSNR [16] and Structural SIMilarity(SSIM) [7] are employed in the action of evaluation in this paper, which has frequently been applied to estimate the quality of colorizing images by using deep neural networks.

#### Peak signal-to-noise ratio (PSNR)

PSNR is normally employed to estimate the quality of a compressed image compared with the original image, which is defined by Mean Squared Error (MSE).

1 $$  PSNR = 10 \cdot log_{10} (\frac{MA{X_{R}^{2}}}{MSE}) = 20 \cdot log_{20}(\frac{MAX_{R}}{\sqrt{MSE}})  $$

where the smaller M*SE*, the higher P*SNR*, the smaller of distortion. This trend means that the generated image has the better quality.

#### SIMilarity (SSIM)

The basic principle is that natural images are highly structured, the adjacent pixels have a strong correlation, this correlation expresses the structural information of visual objects [37]. The range of *SSIM* is 0 to 1. The larger the SSIM value, the higher the image quality. We define two images as **x** and **y**, one is our reference picture, the other is the image obtained after rendering. Based on three comparative measures between samples (**x**) and (**y**): Luminance(**l**), contrast(**c**), and structure(**s**), we calculate the similarity as,

2 $$  SSIM(\mathbf{x}, \mathbf{y}) = [l(\mathbf{x}, \mathbf{y})]^{\alpha} [c(\mathbf{x}, \mathbf{y})]^{\beta} [s(\mathbf{x}, \mathbf{y})]^{\gamma}  $$

**Fig. 9:**
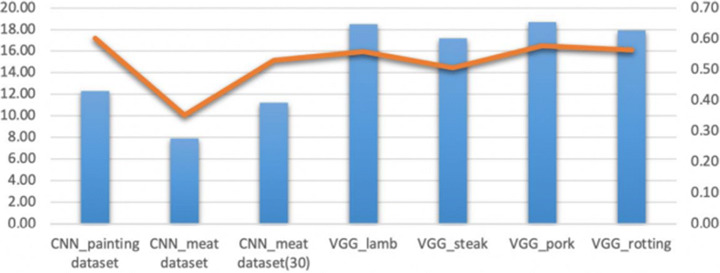
The comparisons of PSNR and SSIM in the colorized COVID-19 lung CT images

**Fig. 10:**
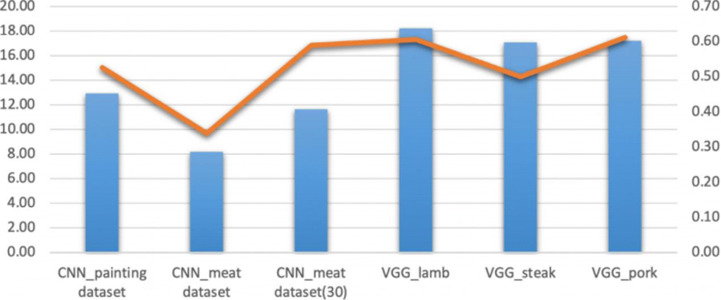
The comparisons of PSNR and SSIM in the healthy lung CT images

In order to discover the most suitable method to colorize the COVID-19 lung CT image and healthy lung CT images, respectively, we deploy the colorizing consequences after applying the reference images, training datasets, and target images (COVID-19 infected lung images and healthy lung images, respectively), corresponding to PSNR and SSIM in Figs. 9 and 10.

The infected lung CT images are used as the target image to produce the colorizing images in Fig. 9. The average PSNR of the hybrid methods is approximately 28.0, while the CNN-based method is nearly 21.0 in PSNR.

Similarly, in general, the healthy lung CT images are employed as the target image for colorization in Fig. 10. The colour lung CT images are formed by using the hybrid colorization approach that are more high quality than the automatic colorization model (CNN-based method). The average value of SSIM in the method of utilizing reference images closes to 0.55. The CNN-based model is designed to apply the meat data composed of 30 images in the CNN-based methods, the SSIM values show that meat data (30 pictures) is more significant than other datasets. The PSNR values reveal the hybrid method via the reference images to generate more trustworthy than the CNN-based method that utilizes the training datasets to train the model.

## Conclusion and future work

Two separated deep networks are applied to our experiments. One is essentially to figure out a fully automatic colorization model based on ResNet network and train three sets of distinctive data to obtain the corresponding outcomes. In order to discover the correlation between the images in the training datasets and the objective lung CT images, we implemented to reduce the size of the images. The operation is also named as feature extractions and then colorized the corresponding pixels in the target lung CT images. Eventually, it is discovered that the consequence of the images generated by using the image data is the most trustworthy. It also demonstrates that the quantity of the data have not direct influence on the finally generated images. Moreover, it witnesses that even fewer data also attains the outcomes after the entire training procedure. Deep neural networks are literately more magnificent than other machine learning methods.

In the second approach, we utilized VGG-19 to construct a deep neural network model so as to extract the content of the reference images and then transfer both features to the target grayscale lung CT images. In order to gain the input reference images, we designed a feature space in accordance with the filter response of each layer of the VGG-19 network to capture textural information. It consists of the correspondence among various filter responses within the spatial domain of the feature map. We find that lamb images as the reference have more beneficial than the rest of meat images because of a few modifications in the original image. Furthermore, the reference image is directly related to the generated lung CT images. Eventually, through comparing these two methods, we discovered that the hybrid method gains more favourable outcomes than the fully automatic method either from a human visual system or scientific computations (PSNR and SSIM).

Overall, the hybrid method with the reference images generated more realistic outcomes compared to other two approaches because the colours of the colorized lung CT images resemble the original human lung colour. Furthermore, from the results of PSNR and SSIM, there are not obvious variations compared to the original images. Naturally, human visual system conforms to our results as the necessary reference.

In future experiments, regarding the configuration of the model, we would like to propose deep neural networks as the training models for colorizing grayscale lung CT images. Meanwhile, we will increase the training samples for various models in terms of quantity and quantity. Wang [29].

## References

[1] Agatonovic-Kustrin S, Beresford R (2000). Basic concepts of artificial neural network (ANN) modeling and its application in pharmaceutical research. J Pharm Biomed Anal.

[2] Aimar A, Mostafa H, Calabrese E, Rios-Navarro A, Tapiador-Morales R, Lungu IA, Milde MB, Corradi F, Linares-Barranco A, Liu SC (2018). Flexible convolutional neural network accelerator based on sparse representations of feature maps. IEEE Trans Neural Netw Learn Syst.

[3] Baldassarre F, Morín DG, Rodés-guirao L (2017) Deep koalarization:, Image colorization using CNNs and Inception-ResNet-v2. arXiv:http://arxiv.org/abs/1712.03400

[4] Bugeau A, Ta VT, Papadakis N (2013). Variational exemplar-based image colorization. IEEE Trans Image Process.

[5] Buzug TM (2011). Computed tomography. Springer handbook of medical technology..

[6] Charpiat G, Hofmann M, Schölkopf B (2008). Automatic image colorization via multimodal predictions. European conference on computer vision..

[7] Chen MJ, Bovik AC (2011). Fast structural similarity index algorithm. J Real-Time Image Proc.

[8] Cheng Z, Yang Q, Sheng B (2015). Deep colorization. IEEE International conference on computer vision.

[9] Chia AYS, Zhuo S, Gupta RK, Tai YW, Cho SY, Tan P, Lin S (2011). Semantic colorization with Internet images. ACM Trans Graph.

[10] Fan DP, Zhou T, Ji GP, Zhou Y, Chen G, Fu H, Shen J, Shao L (2020). Inf-net: Automatic COVID-19 lung infection segmentation from CT images. IEEE Trans Med Imaging.

[11] Galun M, Sharon E, Basri R, Brandt A (2003). Texture segmentation by multiscale aggregation of filter responses and shape elements. IEEE International conference on computer vision..

[12] Gupta RK, Chia AYS, Rajan D, Ng ES, Zhiyong H (2012). Image colorization using similar images. ACM International conference on multimedia..

[13] He M, Liao J, Chen D, Yuan L, Sander PV (2019). Progressive color transfer with dense semantic correspondences. ACM Trans Graph.

[14] Hodnett M, Wiley JF (2018) R Deep learning essentials: A step-by-step guide to building deep learning models using tensorFlow, Keras, and MXNet Packt Publishing Ltd

[15] Ironi R, Cohen-Or D, Lischinski D (2005). Colorization by example. Rendering techniques..

[16] Johnson DH (2006). Signal-to-noise ratio. Scholarpedia.

[17] Larsson G, Maire M, Shakhnarovich G (2016). Learning representations for automatic colorization. European conference on computer vision..

[18] Levin A, Lischinski D, Weiss Y (2004). Colorization using optimization. ACM SIGGRAPH 2004.

[19] Liu X, Wan L, Qu Y, Wong TT, Lin S, Leung CS, Heng PA (2008). Intrinsic colorization. ACM SIGGRAPH Asia..

[20] Luan Q, Wen F, Cohen-Or D, Liang L, Xu YQ, Shum HY (2007). Natural image colorization. Eurographics conference on rendering techniques..

[21] Morimoto Y, Taguchi Y, Naemura T (2009). Automatic colorization of grayscale images using multiple images on the web. ACM SIGGRAPH 2009.

[22] Pan F, Ye T, Sun P, Gui S, Liang B, Li L, Zheng D, Wang J, Hesketh RL, Yang L et al (2020) Time course of lung changes on chest CT during recovery from 2019 novel coronavirus (COVID-19) pneumonia Radiology 10.1148/radiol.2020200370 PMC7233367 32053470

[23] Pandey ATS, Sharma PDN (2019). Image colorization using deep learning. Int J Scientif Res Eng Trends.

[24] Qu Y, Wong TT, Heng PA (2006). Manga colorization. ACM Transactions on Graphics (TOG).

[25] Reinhard E, Adhikhmin M, Gooch B, Shirley P (2001). Color transfer between images. IEEE Comput Graph Appl.

[26] Sỳkora D, Buriánek J, žára J (2004). Unsupervised colorization of black-and-white cartoons. International symposium on non-photorealistic animation and rendering..

[27] Tai YW, Jia J, Tang CK (2005). Local color transfer via probabilistic segmentation by expectation-maximization. IEEE Conference on computer vision and pattern recognition. vol 1..

[28] Verdun F, Racine D, Ott J, Tapiovaara M, Toroi P, Bochud F, Veldkamp W, Schegerer A, Bouwman R, Giron IH (2015). Image quality in CT: from physical measurements to model observers. Physica Medica.

[29] Wang Y (2021). Colorizing Grayscale CT images using deep learning (Masters Thesis).

[30] Welsh T, Ashikhmin M, Mueller K (2002). Transferring color to greyscale images. Annual conference on computer graphics and interactive techniques.

[31] Wu Z, Shen C, Van Den Hengel A (2019). Wider or deeper: Revisiting the resNet model for visual recognition. Pattern Recogn.

[32] Xie S, Girshick R, Dollár P, Tu Z, He K (2017). Aggregated residual transformations for deep neural networks. IEEE Conference on computer vision and pattern recognition..

[33] Yatziv L, Sapiro G (2006). Fast image and video colorization using chrominance blending. IEEE Trans Image Process.

[34] Zhang LJ, Zhou CS, Schoepf UJ, Sheng HX, Wu SY, Krazinski AW, Silverman JR, Meinel FG, Zhao YE, Zhang ZJ (2013). Dual-energy CT lung ventilation/perfusion imaging for diagnosing pulmonary embolism. Eur Radiol.

[35] Zhang R, Isola P, Efros AA (2016). Colorful image colorization. European conference on computer vision. Springer.

[36] Zhang R, Zhu JY, Isola P, Geng X, Lin AS, Yu T, Efros AA (2017) Real-time user-guided image colorization with learned deep priors. arXiv:http://arxiv.org/abs/1705.02999

[37] Zujovic J, Pappas TN, Neuhoff DL (2013). Structural texture similarity metrics for image analysis and retrieval. IEEE Trans Image Process.

